# Emerging viral diseases of Southeast Asia and the Western Pacific.

**DOI:** 10.3201/eid0707.017703

**Published:** 2001

**Authors:** J S Mackenzie, K B Chua, P W Daniels, B T Eaton, H E Field, R A Hall, K Halpin, C A Johansen, P D Kirkland, S K Lam, P McMinn, D J Nisbet, R Paru, A T Pyke, S A Ritchie, P Siba, D W Smith, G A Smith, A F van den Hurk, L F Wang, D T Williams

**Affiliations:** University of Queensland, Brisbane, Australia. jmac@biosci.uq.edu.au

## Abstract

Over the past 6 years, a number of zoonotic and vectorborne viral diseases have emerged in Southeast Asia and the Western Pacific. Vectorborne disease agents discussed in this article include Japanese encephalitis, Barmah Forest, Ross River, and Chikungunya viruses. However, most emerging viruses have been zoonotic, with fruit bats, including flying fox species as the probable wildlife hosts, and these will be discussed as well. The first of these disease agents to emerge was Hendra virus, formerly called equine morbillivirus. This was followed by outbreaks caused by a rabies-related virus, Australian bat lyssavirus, and a virus associated with porcine stillbirths and malformations, Menangle virus. Nipah virus caused an outbreak of fatal pneumonia in pigs and encephalitis in humans in the Malay Peninsula. Most recently, Tioman virus has been isolated from flying foxes, but it has not yet been associated with animal or human disease. Of nonzoonotic viruses, the most important regionally have been enterovirus 71 and HIV.


### Emerging Viral Diseases of Southeast Asia and the Western Pacific

**J.S. Mackenzie,* K.B. Chua,†‡ P.W. Daniels,‡ B.T. Eaton,‡ H.E. Field,§ R.A. Hall,* K. Halpin,*§ C.A. Johansen,*¶ P.D. Kirkland,# S.K. Lam,† P. McMinn,** D.J. Nisbet,* R. Paru,†† A.T. Pyke,‡‡ S.A. Ritchie,¶ P. Siba,†† D.W. Smith,§§ G.A. Smith,‡‡ A.F. van den Hurk,* L.F. Wang,‡ D.T. Williams*‡**

*University of Queensland, Brisbane, Australia; †University of Malaya, Kuala Lumpur, Malaya; ‡CSIRO Australian Animal Health Laboratory, Geelong, Australia; §Animal Research Institute, Brisbane, Australia; ¶Queensland Health Tropical Public Health Unit, Cairns, Australia; #Elizabeth McArthur Agricultural Institute, New South Wales, Australia; **Princess Margaret Hospital for Children, Perth, Australia; ††Papua New Guinea Institute of Medical Research, Goroka, PNG; ‡‡Queensland Health Scientific Services, Brisbane, Australia; §§PathCentre, Nedlands,Western Australia

Over the past 6 years, a number of zoonotic and vectorborne viral diseases have emerged in Southeast Asia and the Western Pacific. Vectorborne disease agents discussed in this article include Japanese encephalitis, Barmah Forest, Ross River, and Chikungunya viruses. However, most emerging viruses have been zoonotic, with fruit bats, including flying fox species as the probable wildlife hosts, and these will be discussed as well. The first of these disease agents to emerge was Hendra virus, formerly called equine morbillivirus. This was followed by outbreaks caused by a rabies-related virus, Australian bat lyssavirus, and a virus associated with porcine stillbirths and malformations, Menangle virus. Nipah virus caused an outbreak of fatal pneumonia in pigs and encephalitis in humans in the Malay Peninsula. Most recently, Tioman virus has been isolated from flying foxes, but it has not yet been associated with animal or human disease. Of nonzoonotic viruses, the most important regionally have been enterovirus 71 and HIV.

With a few exceptions, most interest and attention regarding emerging viral diseases in Southeast Asia and the Western Pacific have been directed at zoonotic and vector-borne diseases. However, other viral diseases have also been prominent and will be mentioned briefly. Enterovirus 71, for example, one of the common causes of hand, foot, and mouth disease, has caused a number of regional epidemics that have included cases of encephalitis. It is also not possible to discuss emerging viruses without briefly referring to HIV infection and AIDS in the region.

Several reviews have described the emergence of viruses in one or more countries in the region (1-8). Building on these earlier papers, this article will provide an up-to-date summary of the major viruses and their recent outbreaks (Figure).

### Vector-borne Viral Disease Agents

Dengue viruses and Japanese encephalitis (JE) viruses are the major vector-borne disease agents in the Asia-Pacific region, with Ross River, Chikungunya, and Barmah Forest viruses important in relatively restricted geographic areas. Dengue viruses cause frequent epidemics throughout the region and are endemic in a number of countries, including Indonesia, Papua New Guinea, Malaysia, Thailand, Cambodia, and Vietnam. Epidemic activity in northeastern Australia and the Pacific island nations is the result of reintroductions by viremic travelers (8). This report will focus

Address for correspondence: John S. Mackenzie, Department of Microbiology and Parasitology, University of Queensland, Brisbane, QLD 4072, Australia; fax: 61-7-3365-4648; email: jmac@biosci.uq.edu.au

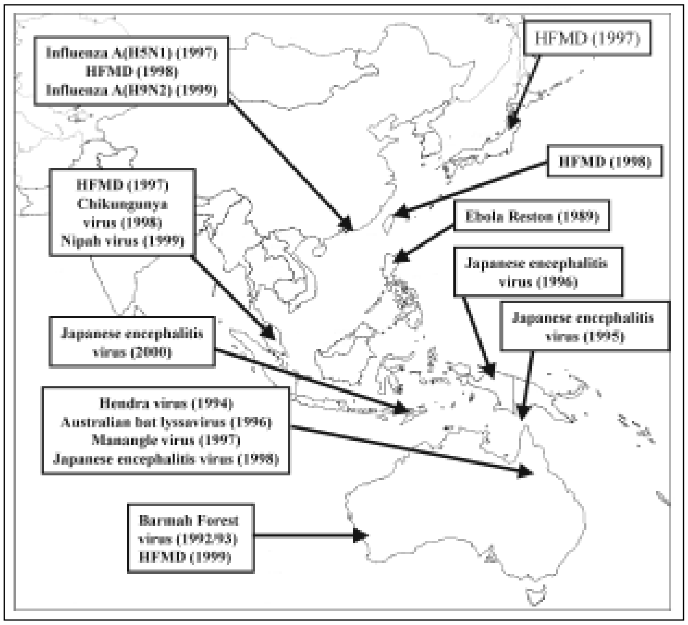

Figure. Outbreaks of viral diseases in Southeast Asia and the Western Pacific over the past decade.

on JE virus, with short descriptions of the Ross River, Chikungunya, and Barmah Forest viruses.

### JE Virus

JE virus is endemic throughout much of Southeast Asia (9), but, with the exception of serologic evidence of activity on Lombok Island and a single isolate from Flores Island (10), it was not known to occur in the Australasian zoogeographic region. However, it emerged in the Torres Strait of northern Australia in 1995 to cause three cases of encephalitis on Badu Island, two of which were fatal (11,12). Ten isolates of JE virus were obtained, two from human serum specimens drawn from subclinical infections and eight from *Culex annulirostris* mosquitoes. Subsequent seroepidemiologic studies showed that JE virus had existed in the Daru area of the Western Province of Papua New Guinea since at least 1989 and that it was spreading rapidly in several provinces. Virus activity has been observed in the northern Torres Strait every year since 1995, except 1999. In 1998, another case of encephalitis occurred on Badu Island (13), and widespread seroconversions were found in sentinel pigs. A total of 43 virus isolates were obtained from *Cx. annulirostris* and one from *Aedes vigilax* mosquitoes on Badu Island.

Virus activity also spread southward into northern mainland Australia; the first clinical case was seen in a fisherman who contracted the infection at the mouth of the Mitchell River in southwestern Cape York. Serologic evidence of infection in pigs indicated that transmission cycles had occurred in two local communities, but no other JE infections were noted in community residents (13). JE virus activity was also observed in northern Cape York in various communities near Bamaga; seroconversions had occurred in sentinel pigs, and JE virus was isolated from three of the pigs. Once again, no evidence of subclinical human infections was found in communities near Bamaga. No isolates of JE virus were obtained from pools of *Cx. annulirostris* collected at various sites in Cape York, even though the number of mosquitoes processed was equal to the number of those from Badu Island (14). The epidemic activity of JE virus on Badu Island in 1995 had been driven by the very close proximity of domestic pigs, mosquito breeding sites, and human habitation (11), and this almost certainly was true again in 1998.

After the 1998 case, a new communal piggery was constructed about 3 km from the community. In 2000, further JE virus activity was found on Badu Island with sentinel pig seroconversions, three of which yielded virus isolates; one virus isolate was obtained from *Cx. gelidus* mosquitoes, but no human cases occurred. The absence of human cases may have been due to the widespread use of JE vaccine, which contained inactivated virus, in the central and northern Torres Strait islands and may also have been associated with the move of domestic pigs away from backyards to the communal piggery. The isolation of virus from *Cx. gelidus*, a major vector of JE virus in Southeast Asia, was particularly important because this species of mosquito had not previously been recognized in Australia (it had earlier been identified as the closely related *Cx. vicinus*) (15). Recently, however, *Cx. gelidus* has become established at a number of sites across northern Australia (P. Whelan, S.A. Ritchie, unpub. data). This reinforced concern about the potential for the virus to spread across Australia where suitable vectors and vertebrate hosts are plentiful (16,17).

The role of marsupials as possible vertebrate hosts remains to be determined. However, some preliminary bloodmeal studies indicate that the most prevalent mosquito vector, *Cx. annulirostris*, prefers to feed on wallabies and other marsupials rather than feral pigs (A. Van Den Hurk, pers. comm.). Under experimental conditions, wallabies do not appear to be viable hosts because of the low level of viremia elicited by JE infection (P. Daniels, unpub. data).

JE virus occurs widely in the Western Province of Papua New Guinea as evidenced by virus isolations taken from *Cx. sitiens* group mosquitoes; a number of clinical cases of encephalitis, and seropositive humans and pigs over a wide area (17-19). Serologic evidence also suggests that JE virus has spread from the Western Province into the Southern Highlands and Gulf Provinces (17) and that it has emerged in the West Sepik Province in the north and been responsible for outbreaks of encephalitis on Normanby Island and at Alatau in Milne Bay Province in the eastern part of the country. A probable human case of JE infection has also been reported from Irian Jaya (20), and antibodies to JE have been found in human serosurveys there (21). The rapidity with which JE virus has spread through Papua New Guinea places some nearby Pacific nations, such as the Solomon Islands and Vanuatu, at risk.

Molecular phylogenetic studies have clearly demonstrated that the JE virus that spread into the Torres Strait in 1995 originated in Papua New Guinea. Indeed, all JE virus isolates from the 1995 incursion, as well as those from Cape York and the Torres Strait in 1998, were almost identical to each other and to three isolates from Papua New Guinea and were most closely related to JE strains from Malaysia, southern Thailand, and Indonesia (unpub. data) (13,15,17,19,22). In addition, these viruses shared an 11-base deletion in the 3' untranslated regions (UTR) immediately downstream from the termination codon of the virus's single open-reading frame (23). The virus isolates obtained from pig sera and from *Cx. gelidus* mosquitoes collected on Badu Island in 2000, however, showed considerable variation from previous isolates. Although they retained the 11-base deletion in the 3′ UTR, their nucleotide sequences differed markedly in the prM gene, E gene, and the NS5-3UTR regions of the genome. The Badu 2000 isolates appear to be phylogenetically more closely related to viruses from Cambodia, northern Thailand, and Korea (A.T. Pyke, D.T. Williams, D.J. Nisbet, A.F. van den Hurk, C.T. Taylor, C.A. Johansen, unpub. data).

The direction and mechanism of the spread of JE virus from the oriental zoogeographic zone to the Australasian zoogeographic zone remain unknown (10,17). The most likely mechanism, however, is the gradual spread in mosquito-bird and mosquito-pig transmission cycles across the eastern Indonesian archipelago from Bali in the west to Irian Jaya in the east. In support of this theory, antibodies to JE virus were found in sera taken from pigs at various sites, including Timor and Jayapura (24), and the very recent serologic diagnosis of clinical cases of JE in Timor (L. Hueston, unpub. data). Computer simulation suggests that low pressure systems west of the Torres Strait/Cape York area produce strong northerly winds that could carry infected mosquitoes from the

New Guinea mainland to the Torres Strait and Cape York Peninsula (S.A. Ritchie, W. Rochester, unpub. data).

### Barmah Forest Virus

Barmah Forest (BF) virus is an alphavirus that is enzootic to Australia. It circulates among mosquitoes and terrestrial animals, especially certain marsupial species (25,26) and causes an epidemic polyarthritis-like disease known as Barmah Forest virus disease (27,28). Human infection with BF virus has been recognized since 1986 and its incidence has increased since then, at least partly as a result of greater clinical awareness and availability of diagnostic reagents. From its endemic foci in northern and eastern Australia, the virus has spread into other geographic areas during the past decade, causing epidemics in north, northwest, and southwest Australia and recently in southeast Australia (28-30), establishing a low level endemic pattern with a relatively stable number of reported cases since BF disease became reportable in Australia in 1995 (Table). However, human disease due to BF virus in southern Australia or Tasmania has not yet been confirmed.

### Ross River Virus

Ross River (RR) virus causes an epidemic polyarthritis (27,28), and, to avoid confusing it with other viruses that cause similar symptoms, the disease is now referred to as Ross River virus disease. It is found in all Australian states and territories, as well as Papua New Guinea. Serologic evidence indicates that it also occurs in the Solomon Islands. The concept that RR virus is an emerging disease is somewhat difficult to justify, however. Although the number of reported cases of RR virus disease has increased slightly during the past decade (Table), this increase can be largely attributed to a combination of improved diagnostic reagents, greater awareness by clinicians, a trend towards new housing developments in coastal regions adjacent to salt marsh wetlands, and changing demographics as people migrate northward to warmer climates in retirement. However, RR virus clearly has the potential to spread, as demonstrated by the very extensive outbreak in Pacific Island nations in 1979-80 (25).

### Chikungunya Virus

Chikungunya virus was relatively common in southern and southeastern Asia in the 1960s. After causing outbreaks in India, Sri Lanka, Burma, and Thailand, it all but disappeared in India, Sri Lanka, Burma (31), and Bangkok (32). However, localized outbreaks and sporadic cases continued in Burma, Thailand, and the Philippines in the 1980s. In addition, the virus spread into Indonesia for the first time from 1982 to 1985, with outbreaks in South Sumatra, Java, and West Kalimantan (1982); southern, eastern, and central Kalimantan (1983); southern Sulawesi (1993); eastern Timor and eastern Nusatengarra (1984); Mollucas Islands (1985); North Sulawesi (1985); and Irian Jaya (1985 to 1986) (33). Outbreaks occurred in Thailand in 1995 (34) and Malaysia in 1998 to 1999 (S.K. Lam, K.B. Chua, D.W. Smith, unpub. data). The latter was the first outbreak to be recorded in Malaysia, although in the 1960s, antibody to Chikungunya virus was relatively common in the people of Malay Peninsula and Sarawak. The outbreak involved 51 confirmed cases in a densely populated, urban area near Kuala Lumpur. The major symptoms were fever (2 to 5 days), transient maculopapular rash on the trunk and limbs (2 to 3 days), and severe back pain. About 80% of patients had some form of joint symptoms, either arthralgia or arthritis, involving the small joints of hands and feet.

### Emerging Zoonotic Viruses

A number of viruses have emerged from fruit bats (flying foxes), particularly members of the genus *Pteropus*, over the past 6 years. These viruses include Hendra and Nipah, two members of a new genus within the Paramyxoviridae; Menangle and Tioman viruses, two new members of the *Rubulavirus* genus in the family Paramyxoviridae; and Australian bat lyssavirus, a member of the *Lyssavirus* genus in the family Rhabdoviridae, closely related to classic rabies virus.

### Hendra Virus

In September 1994, a sudden outbreak of an acute respiratory syndrome occurred among thoroughbred horses in a training complex in Brisbane, Australia; 13 horses and their trainer died. The causal agent, a previously undescribed member of the family Paramyxoviridae, was initially named equine morbillivirus (35), but was renamed Hendra virus (after the Brisbane suburb where the outbreak occurred). A second (apparently unrelated) outbreak resulted in the death of two horses and their owner near Mackay, nearly 1000 km north of Brisbane(36-38). The outbreak preceded the events at Hendra and was retrospectively identified in 1995. Most recently, a single fatal equine case occurred near Cairns in North Queensland in January 1999 (39,40).

To evaluate the theory that Hendra virus existed in a wildlife reservoir, serologic surveillance of wildlife species was undertaken, and, in April 1996, anti-Hendra virus antibodies were identified in a black flying fox (*Pteropus alecto)*. Within weeks, evidence of infection was found in the other three species of Australian flying foxes; gray-headed flying fox (*P. poliocephalus*), little red flying fox (*P. scapulatus*), and spectacled flying fox (*P. conspicillatus*) (41). In 1996, a Hendralike virus was isolated from the reproductive tract of a seemingly healthy, pregnant gray-headed flying fox. A range of tests showed the bat isolate to be indistinguishable from

Table. Annual case notifications of Ross River (RRV) and Barmah Forest (BFV) virus infections, 1991–1999

| | 1991 | 1992 | 1993 | 1994 | 1995 | 1996 | 1997 | 1998 | 1999 |
|---|---|---|---|---|---|---|---|---|---|
| RRV | 3,352 | 5,630 | 5,428 | 3,960 | 2,602 | 7,823 | 6,686 | 3,094 | 4,407 |
| BFV | N/A | N/A | N/A | N/A | 736 | 837 | 704 | 558 | 628 |

N/A: not available (BFV was not made a notifiable disease until 1995).
Communicable Diseases Network—Australia, New Zealand—National Notifiable Diseases Surveillance System, pers. comm.

the Hendra virus isolated from horses (42). However, no evidence of illness exists in flying foxes infected naturally (K. Halpin, unpub. data) or infected experimentally (43,44) that can be attributed to infection with Hendra virus, supporting epidemiologic evidence (H.E. Field, unpub. data) that flying foxes are the probable hosts of Hendra virus.

Hendra virus does not appear to be very contagious, and there has been no evidence of infection in humans even in those who have had close contact with injured bats (45). Transmission from flying foxes to horses has not been demonstrated; however, studies done on different species infected experimentally and flying foxes and horses infected naturally have indicated possible modes of transmission. Virus has been isolated from the kidney, urine, and (less so) oral cavity of horses and from the kidney and urine of cats experimentally infected with Hendra virus. Horses have been experimentally infected by the naso-oral route, and cat-to-cat transmission and suspected cat-to-horse transmission have been reported (43,46).

Biologically and genetically, Hendra virus differs significantly from other members of the Paramyxoviridae family. They show morphologic differences, seen in two distinct lengths of surface projections (47), and genetic differences, demonstrated by the genome size. The genome is longer (18,234 nucleotides) than those of members of the *Respirovirus* and *Morbillivirus* genera because it has longer intergenic noncoding sequences and a larger L protein gene (48). These differences, together with limited homology to other members of Paramyxoviridae, indicate that Hendra virus should be classified as the first member of a new genus in this family; the name *Henipavirus* has been suggested (48).

### Nipah Virus

A major outbreak of disease in pigs and humans in the Malay Peninsula from September 1998 to April 1999 resulted in 265 infected persons, 105 of whom died (49), and the eventual destruction of about 1.1 million pigs. The disease in pigs was highly contagious and symptoms included acute fever, respiratory problems, and neurologic signs in infected pigs of all ages. The predominant clinical syndrome in humans was encephalitic rather than respiratory, with clinical signs including fever, headache, myalgia, drowsiness, and disorientation, sometimes proceeding to a coma within 48 hours (50,51). Most infected persons had a history of direct contact with live pigs, and most were pig farmers. Epidemiologic evidence suggested that the disease had been spread primarily by pigs that were transported between farms or to other regions. The primary mode of transmission on pig farms was believed to be through the respiratory route, and this was subsequently confirmed with experiments (52). Investigations have revealed that the virus has caused disease in pigs in Peninsular Malaysia since late 1996. Eleven cases of encephalitis and pneumonia resulting from Nipah virus infection also occurred in Singapore during the outbreak in Malaysia; one abattoir worker who worked on pigs imported from Malaysia died (53).

Preliminary research on the new virus, subsequently named Nipah virus, revealed that it had ultrastructural, antigenic, serologic, and molecular characteristics similar to Hendra virus (49). Molecular studies confirmed that Nipah virus was closely related to Hendra virus, with specific genes sharing 70% to 88% nucleotide homologies and 67% to 92% amino acid homologies, and with identical intergenic regions and nearly identical gene start-and-stop sequences (54). Thus, these two viruses are members of a new proposed genus within the family Paramyxoviridae (48).

Surveillance of wildlife species for evidence of the origin of Nipah virus was an integral part of the outbreak investigation (55). Knowing the similarities between Nipah virus and Hendra virus, attention was focused on surveillance of bats. In common with most countries in Southeast Asia, Peninsular Malaysia has a great diversity of bat species: at least 13 species of fruit bat (suborder Megachiroptera), including two flying fox species, and at least 60 species of insectivorous bats (suborder Microchiroptera) (56).

Antibodies that neutralize Nipah virus were found in 21 bats from five species (four species of fruit bat, including two flying fox species and one insectivorous species) (J. M. Yob, H.E. Field, unpub. data). Cross-neutralization of Nipah antigen by antibodies to Hendra virus was excluded as the cause of reactivity. Attempts to detect the virus in sera using both culture and amplification of RNA in reverse transcriptase-polymerase chain reaction were unsuccessful. However, Nipah virus has recently been isolated from the urine of flying foxes (K.B. Chua, S.K. Lam, unpub. data).

### Menangle and Tioman Viruses

A previously undescribed virus, Menangle virus, was isolated from stillborn piglets with deformities at a large commercial piggery in New South Wales (57). The virus was responsible for a reduced farrowing rate and for causing the stillbirths with deformities. The affected stillborn piglets frequently showed severe degeneration of the brain and spinal cord, arthrogryposis, brachygnathia, and, occasionally, fibrinous body cavity effusions and pulmonary hypoplasia. Virus was isolated from lung, brain, and heart tissues of infected piglets and shown to be morphologically similar to viruses in the family Paramyxoviridae. No disease was seen in postnatal animals of any age, but a high proportion of serum specimens (>90%) collected from animals of all ages contained high titers of antibodies that neutralized the virus. Phylogenetic studies with nucleotide sequences generated from cDNA of Menangle virus showed that the virus was a member of the *Rubulavirus* genus within the family Paramyxoviridae and unrelated to any other virus known to infect pigs. Convalescent-phase serum samples from two persons who worked on pigs were found to have high titers of antibodies that neutralized the new virus. Both workers had an influenzalike illness with a rash during the pig outbreak, and extensive serologic testing showed no evidence of any alternative cause. Therefore, the illness was likely caused by the Menangle virus (58).

Notably, a large breeding colony of gray-headed and little red flying foxes roosted within 200 m of the affected piggery. In a preliminary study, 42 of 125 serum samples collected from the bats had antibodies that neutralized the new virus. In addition, antibodies were found in sera collected in 1996, before the outbreak, and from a colony of flying foxes 33 km from the piggery (57). Thus, flying foxes were likely the primary hosts of the virus that caused the outbreak. All other sera collected from a variety of wild and domestic animals in the vicinity of the affected piggery tested seronegative for the virus.

The search for the natural host of Nipah virus led to the discovery of another new member of the Paramyxoviridae family, Tioman virus, which was isolated from the urine of flying foxes (*P. hypomelanus*) and found on Tioman island off the eastern coast of the Malay Peninsula (K.B. Chua, unpub. data). Electron microscopic analysis of virus-infected cells revealed spherical and pleomorphic enveloped virus particles (100 nm to 350 nm) compatible in structure with those of viruses in the family Paramyxoviridae. Tioman virus failed to react with antibodies against a number of known Paramyxoviridae members but did cross-react in immunofluorescence tests with antisera to Menangle virus. However, antiserum to Menangle virus failed to neutralize Tioman virus. To characterize the molecular structure of Tioman virus, a cDNA subtraction strategy that isolated virus-specific cDNA from virus-infected cells was employed. Complete gene sequences for the nucleocapsid protein (N) and phosphoprotein (P/V) have been determined and recombinant N protein produced in baculovirus. The recombinant Tioman virus N and V proteins reacted with porcine antisera to Menangle virus in Western blots, confirming the serologic cross-reactivity observed during initial virus characterization. Phylogenetic analysis indicated that Tioman and Menangle viruses are closely related members of the *Rubulavirus* genus. Sequences of the nucleocapsid protein gene of the two viruses are approximately 70% identical at the nucleotide level and approximately 85% identical at the amino acid level (K.B. Chua, L.F. Wang, unpub. data). The potential of Tioman virus to cause disease in animals and humans is unknown. Its relationship with Menangle virus highlights the need to determine not only if it will replicate and cause disease in pigs, but also if pigs can act as amplifying hosts and transmit the disease to other species, as appears to be the case with Menangle virus.

### Australian Bat Lyssavirus

Australian bat lyssavirus (ABLV) was first discovered in a black flying-fox bat (*P. alecto*) in Ballina in northern New South Wales that was displaying neurologic signs (59). ABLV has since been discovered in all four species of flying-fox bats (black, gray-headed, little red, and spectacled) throughout their geographic range (H.E. Field, unpub. data) and in an insectivorous bat (yellow-bellied, sheath-tail, *Saccolaimus flaviventris* species) in Queensland. The virus was antigenically similar to classic rabies virus and therefore a member of lyssavirus serotype 1, but its genetic sequence was distinguishable and was therefore ascribed a new genotype number—genotype 7 (60). Further research has shown that two closely related, but genetically distinguishable, strains of ABL occur in Australia, one in flying fox bats and the other in insectivorous bats. Researchers at the Centers for Disease Control and Prevention have found that rabies vaccine may elicit a protective immune response to ABLV (61,62), and vaccination is now offered to all those at risk of exposure. The first human case of ABLV infection occurred in 1996; a 39-year-old animal handler, who had been scratched and possibly bitten 5 weeks earlier by a yellow-bellied sheath-tailed bat, died of encephalitis (63). The second case, also manifested by fatal encephalitis, occurred in a 27-year-old woman who had been bitten by a flying fox more than 2 years previously (64). In both instances, the clinical signs were consistent with classic rabies infection. The incidence of ABLV infection in bats is unknown. In one study, about 6% of sick, injured, or orphaned bats were antibody-positive for ABLV (65). However, antibodies have also been found in apparently healthy bats (P.W. Daniels, R. Lunt, H.E. Field, unpub. data), but the role that these bats play in the ecology of ABLV, while of concern, remains to be elucidated. Virus isolations from bats, however, have generally come from animals exhibiting behavioral or neurologic signs. Most infected bats appear depressed, although some exhibit aggressive behavior (66). Histopathologic examinations of infected bats have been carried out, and in most bats the lesions found were nonsuppurative meningoencephalitic and ganglioneuritic in nature, similar to that seen in rabies, except that the number of Negri bodies was variable (65). Immunoperoxidase tests showed lyssaviral antigen was variable in intensity and distribution. Reactions did not always occur in the salivary glands, even if virus was present in the brain (67).

The finding of ABLV in Australian frugivorous and insectivorous bats has had major public health implications: people at risk for exposure must be vaccinated, and those with suspected infection must undergo expensive postexposure prophylaxis (65). Indeed, flying foxes are common in urban areas of eastern and northern Australia; many towns and cities are home to colonies of many hundreds.

### Other Viral Diseases

### Enterovirus 71 Infection

Enterovirus 71 (EV71) infection manifests most frequently as a mild childhood illness known as hand, foot, and mouth disease (HFMD) and is clinically indistinguishable from HFMD caused by coxsackievirus type A16 (CA16). However, EV71 has a propensity to cause severe neurologic disease during acute infection (68,69), a feature not observed in CA16 infections. Children under 4 years of age are particularly susceptible to the most severe forms of EV71-associated neurologic disease, including meningitis, brainstem or cerebellar encephalitis (or both), and poliomyelitis-like paralysis. The neurologic complications of EV71 infection may occasionally cause permanent paralysis or death.

Since 1997, several large epidemics of EV71 infection have been reported in East and Southeast Asia and Australia. The first epidemic occurred in 1997 in Sarawak (70), followed by smaller outbreaks in Singapore, Japan (71) and the Malay Peninsula (72). These outbreaks were associated with numerous cases of HFMD in young children and were accompanied by neurologic complications such as aseptic meningitis, poliomyelitislike paralysis, and cerebellar ataxia in a small number of cases. However, a syndrome of rapidly fatal neurogenic pulmonary edema and hemorrhage was also observed during these outbreaks (73,74). Thirty-four deaths occurred in Sarawak as a result of this disease (70); four deaths were reported in Kuala Lumpur (72) and three in Japan. In 1998, the largest recorded epidemic of EV71-associated HFMD occurred in Taiwan (75,76), involving the whole island, with approximately 130,000 cases of HFMD reported. There were 405 cases of severe neurologic disease and 78 cases of fatal neurogenic pulmonary edema (75). A small outbreak was also reported in Hong Kong at the same time.

The most recent large outbreak of EV71 infection occurred in Perth, Australia, in 1999 (77). Numerous cases of HFMD were reported over a 6-month period (March to August), and 29 cases of severe neurologic disease were diagnosed. The spectrum of neurologic disease seen in Perth included aseptic meningitis, acute cerebellar ataxia, and acute flaccid paralysis; however, no cases of fatal neurogenic pulmonary edema were observed.

Before the large outbreaks of EV71 infection in the Asian-Australasian region, only one case of brainstem encephalitis and neurogenic pulmonary edema due to enterovirus 71 infection had been described (78). Several postmortem studies on those who died of neurogenic pulmonary edema have been published (70,72,79,80). In each case, disease appears to be confined to the brainstem, with histologic evidence of acute inflammatory encephalitis and isolation of EV71 or identification of EV71 antigen within neurons. These studies strongly suggest that pulmonary edema and hemorrhage are of neurogenic origin and secondary to brainstem encephalitis. These findings are supported by neuroradiologic evidence of brainstem pathology in many people who died of fulminant pulmonary edema (71,79,81).

Despite radiologic and histologic evidence of brainstem encephalitis in people who died of neurogenic pulmonary edema and immunohistochemical evidence of the direct involvement of EV71 in brainstem encephalitis, the cause of death in children who contracted the disease during the 1997 outbreak in Sarawak remains controversial. Although many children died as a result of rapidly progressive pulmonary edema (70) similar to that observed elsewhere, a clinical diagnosis of acute myocarditis was made in many cases. In addition, both EV71 and a novel group B adenovirus were isolated from specimens from sterile sites (including brain and heart) and nonsterile sites taken both before and after patients' deaths. The authors suggest that this adenovirus might have played a causative role in these fatal cases either as the primary pathogen or by interacting with EV71. Unfortunately, the data currently available in published literature do not allow a rigorous assessment of the role of adenovirus in this syndrome. Review of additional published postmortem studies done in Sarawak will be necessary to clarify this issue.

Several reports on the molecular epidemiology of recent EV71 activity in Asia have been published (82-85). Unfortunately, the data in these reports cannot be compared directly as different parts of the viral genome were analyzed in these studies. However, all three studies indicate that at least four genetic lineages of EV71 have circulated in Asia since 1997. In addition, there does not appear to be a single neurovirulent genotype associated with severe and fatal cases because three distinct genotypes have been isolated from people who died as a result of infection with EV71 in Sarawak, Peninsular Malaysia and Taiwan. The EV71 outbreak in Western Australia was caused by two distinct genetic lineages of the organism, determined by using VP1 gene-sequencing (P.C. McMinn, unpub. data). The predominant genotype, which was associated with HFMD and some cases of aseptic meningitis, was most closely related to the genotype of viruses isolated in Sarawak during 1997 (>98% nucleotide homology), suggesting a direct link between the two epidemics. The second, minor genotype, which was associated with severe neurologic disease (acute flaccid paralysis, cerebellar ataxia), was most closely related to the genotypes of EV71 strains isolated in Victoria (eastern Australia) in 1995 (>96% nucleotide homology).

Thus, EV71 activity has increased markedly in the Asia-Pacific region during the past 4 years. In addition, a new clinical manifestation of EV71 infection, a rapidly fatal syndrome of neurogenic pulmonary edema associated with brainstem encephalitis, has been identified. Molecular genetic studies of EV71 isolates have indicated that several distinct viral genotypes circulated in Sarawak, Peninsular Malaysia, Japan, Taiwan, and Western Australia between 1997 and 2000, but, unfortunately, it has not yet been possible to show an association between a particular viral genotype and the development of fatal brainstem encephalitis.

### HIV Infection and AIDS

Although discussion of HIV infection and AIDS in Southeast Asia and the Western Pacific region is beyond the scope of this short review, a few comments need to be made about increasing incidence as a component of disease emergence. Two countries in the region, Cambodia and Papua New Guinea, are of particular concern because of the high and increasing incidence of HIV infection, primarily through heterosexual transmission (86). Indeed, Cambodia has the most serious HIV epidemic situation in the region, with the highest HIV infection rate in Asia—3.3% of the most sexually active population (ages 15 to 49). In Papua New Guinea, the prevalence rate is believed to be about 0.6% in the most sexually active population and growing alarmingly, but this figure refers only to the capital city, Port Moresby. Little is known of the prevalance elsewhere, although the second largest city, Lae, probably has a prevalance similar to that of Port Moresby. There is also evidence of increasing HIV infection among people who live along major highways from Lae, especially the highway to Goroka and the Highlands. In addition, because of the very high mobility of the Papua New Guinea population, people in many remote communities have contracted AIDS or HIV infection (M.P. Alpers, pers. comm.). There is also a high incidence of HIV infection among those who inject drugs and increasing heterosexual transmission of HIV in China and Vietnam.

Most emerging diseases in the Asia-Pacific region are due to either novel zoonotic viruses or to an increased incidence or geographic spread of known viruses. The importance of the emergence of novel zoonotic diseases from wildlife cannot be overemphasized. Currently, very few countries anywhere have active wildlife diseases surveillance, but it is hoped that such surveillance activities will eventually increase.

Dr. Mackenzie is a professor and head of the Department of Microbiology and Parasitology, University of Queensland, Brisbane, Australia. His research interests include the epidemiology, ecology, and molecular biology of mosquito-borne and emerging zoonotic viruses.

### References

1. Banerjee K. Emerging viral infections with special reference to India. Indian J Med Res 1996;103:177-200.
2. Beaman MH. Emerging infections in Australia. Ann Acad Med Singapore 1997;26:609-15.
3. Lam SK. Emerging infectious diseases-Southeast Asia. Emerg Infect Dis 1998;4:145-7
4. Corwin A, Simanjuntak CH, Ansari A. Emerging disease surveillance in Southeast Asia. Ann Acad Med Singapore 1997;26:628-31.

5. Mackenzie JS. Emerging viral diseases: some comments from a regional perspective. P N G Med J 1998;41:1-6.
6. Mackenzie JS, Bolton W, Cunningham AL, Frazer IH, Gowans EJ, Grohmann GS, et al. Emerging viral diseases of humans: an Australian and New Zealand perspective. In: Asche V, editor. Recent advances in microbiology. Vol 5. Melbourne: Australian Society for Microbiology Inc; 1997. p. 13-130.
7. Siau H, Yuen KY, Peiris JS, Wong SS, Ho PL, Cao L. et al. Emerging pathogens: the Hong Kong experience. Chin Med J 1997;110:560-6.
8. Mackenzie JS. Emerging viral diseases: an Australian perspective. Emerg Infect Dis 1999;5:1-8.
9. Vaughn DW, Hoke CH. The epidemiology of Japanese encephalitis: prospects for prevention. Epidemiol Rev 1992;14:197-221.
10. Mackenzie JS, Poidinger M, Phillips D, Johansen C, Hall RA, Hanna J, et al. Emergence of Japanese encephalitis virus in the Australasian region. In: Saluzzo JF, Dodet B, editors. Factors in the emergence of arboviral diseases. Paris: Elsevier; 1997. p. 191-201.
11. Hanna J, Ritchie S, Phillips DA, Shield J, Bailey MC, Mackenzie JS, et al. An outbreak of Japanese encephalitis in the Torres Strait, Australia, 1995. Med J Aust 1996;256-60.
12. Ritchie SA, Phillips D, Broom A, Mackenzie J, Poidinger P, van den Hurk A. Isolation of Japanese encephalitis virus from *Culex annulirostris* mosquitoes in Australia. Am J Trop Med Hyg 1997;56:80-4.
13. Hanna JN, Ritchie SA, Phillips DA, Lee JM, Hills SL, van den Hurk AF, et al. Japanese encephalitis in North Queensland, 1998. Med J Aust 1999;170:533-6.
14. Van den Hurk AF, Johansen CA, Mackenzie JS, Zborowski P, Phillips DA, Pyke AT, et al. Flaviviruses isolated from mosquitoes collected during the first recorded outbreak of Japanese encephalitis virus on Cape York Peninsula, Australia. Am J Trop Med Hyg; 2001;64:125-30.
15. Muller MJ, Montgomery BL, Ingram A, Ritchie SA. First records of *Culex gelidus* from Australia. J Am Mosq Control Assoc 2001;17:79-80.
16. Mackenzie JS. Japanese encephalitis: an emerging disease in the Australian region, and its potential risk to Australia. In: Kay BH, Brown MD, Aaskov JG, editors. Arbovirus research in Australia. Vol 7. Brisbane: Queensland Institute of Medical Research; 1997. p. 166-70.
17. Mackenzie JS. The ecology of Japanese encephalitis virus in the Australasian region. Clin Virol (Japan) 1999;27:1-17.
18. Johansen C, Ritchie S, van den Hurk A, Bockarie M, Hanna J, Phillips D, et al. The search for Japanese encephalitis virus in the Western province of Papua New Guinea. In: Kay BH, Brown MD, Aaskov JG, editors. Arbovirus research in Australia. Vol 7. Brisbane: Queensland Institute of Medical Research; 1997. p. 131-6.
19. Johansen CA, van den Hurk AF, Ritchie SA, Zborowski P, Nisbet D, Paru R, et al. Isolation of Japanese encephalitis virus from mosquitoes (Diptera: Culicidae) collected in the Western Province of Papua New Guinea, 1997-1998. Am J Trop Med Hyg 2000;62:631-8.
20. Spicer PE. Japanese Encephalitis in Western Irian Jaya. J Travel Med 1997;4:146.
21. Spicer PE, Phillips D, Pike A, Johansen C, Melrose W, Hall RA. Antibodies to Japanese encephalitis virus in human sera collected from Irian Jaya. Follow-up of a previously reported case of Japanese encephalitis in that region. Trans R Soc Trop Med Hyg 1999;93:511-4.
22. Williams DT, Wang LF, Daniels PW, Mackenzie JS. Molecular characterization of the first Australian isolate of Japanese encephalitis virus, the FU strain. J Gen Virol 2000;81:2471-80.
23. Poidinger M, Hall RA, Mackenzie JS. Molecular characterization of the Japanese encephalitis serocomplex of the *Flavivirus* genus. Virology 1996;218:417-21.
24. Daniels PW, Ginting N. Animal disease surveillance in eastern Indonesia. Report prepared for the Australian Quarantine and Inspection Service and the Office for International Cooperation, Ministry of Agriculture, Indonesia. Bogor: Indonesia International Animal Science Research and Development Foundation, 1992.
25. Mackenzie JS, Lindsay MD, Coelen RJ, Broom AK, Hall RA, Smith DW. Arboviruses causing human disease in the Australasian zoogeographic region. Arch Virol 1994;136:447-67.
26. Mackenzie JS, Broom AK, Hall RA, Johansen CA, Lindsay MD, Phillips DA, et al. Arboviruses in the Australian region, 1990 to 1998. Commun Dis Intell 1998;22:93-100.
27. Mackenzie JS, Smith DW. Mosquito-borne viruses and epidemic polyarthritis. Med J Aust 1996;164:90-2.
28. Flexman JP, Smith DW, Mackenzie JS, Fraser RE, Bass S, Hueston L, et al. A comparison of the diseases caused by Ross River virus and Barmah Forest virus. Med J Aust 1998;169:159-63.
29. Lindsay MDA, Johansen CA, Broom AK, Smith DW, Mackenzie JS. Emergence of Barmah Forest virus in Western Australia. Emerg Infect Dis 1995;1:22-6.
30. Lindsay MD, Johansen CA, Smith DW, Wallace MJ, Mackenzie JS. An outbreak of Barmah Forest virus disease in the southwest of Western Australia. Med J Aust 1995;162:291-4.
31. Pavri K. Disappearance of Chikungunya virus from India and South-East Asia. Trans R Soc Trop Med Hyg 1986;80:491.
32. Burke DS, Nisalak A, Nimmannitya S. Disappearance of Chikungunya virus from Bangkok. Trans R Soc Trop Med Hyg 1985;79:419-20.
33. Suharyono W. Outbreak of Chikungunya virus in Indonesia 1982-1985. Virus Information Exchange Newsletter 1986;3:91.
34. Thaikruea L, Charearnsook O, Reanphumkarnkit S, Dissomboon P, Phonjan R, Ratchbud S, et al. Chikungunya in Thailand: a re-emerging disease? Southeast Asian J Trop Med Public Health 1997;28:359-64.
35. Murray K, Selleck P, Hooper P, Hyatt A, Gould A, Gleeson LJ, et al. A morbillivirus that caused fatal disease in horses and humans. Science 1995;268:94-7.
36. Rogers RJ, Douglas IC, Baldock FC, Glanville RJ, Seppanen KT, Gleeson LJ, et al. Investigation of a second focus of equine morbillivirus infection in coastal Queensland. Aust Vet J 1996;74:243-4.
37. Hooper PT, Gould AR, Russell GM, Kattenbelt JA, Mitchell G. The retrospective diagnosis of a second outbreak of equine morbillivirus infection. Aust Vet J 1996;74:244-5.
38. O'Sullivan JD, Allworth AM, Paterson DL, Snow TM, Boots R, Gleeson LJ, et al. Fatal encephalitis due to novel paramyxovirus transmitted from horses. Lancet 1997;349:93-5.
39. Field H, Barratt P, Hughes R, Shield J, Sullivan N. A fatal case of Hendra virus infection in a horse in north Queensland: clinical and epidemiological features. Aust Vet J 2000;78:279-80.
40. Hooper PT, Gould AR, Hyatt AD, Braun MA, Kattenbelt JA, Hengstberger SG, et al. Identification and molecular characterization of Hendra virus in a horse in Queensland. Aust Vet J 2000;78:281-2.
41. Young PL, Halpin K, Selleck PW, Field H, Gravel JL, Kelly MA, et al. Serologic evidence for the presence in *Pteropus* bats of a paramyxovirus related to equine morbillivirus. Emerg Infect Dis 1996;2:239-40.
42. Halpin K, Young P, Field H, Mackenzie J. Isolation of Hendra virus from pteropid bats: a natural reservoir of Hendra virus. J Gen Virol 2000;81:1927-32.
43. Williamson M, Hooper P, Selleck P, Gleeson LJ, Daniels PW, Westbury HA, et al. Transmission studies of Hendra virus (equine morbillivirus) in fruit bats, horses, and cats. Aust Vet J 1998;76:813-8.
44. Williamson M, Hooper P, Selleck P, Westbury H, Slocombe R. The effect of Hendra virus on pregnancy in fruit bats and guinea pigs. J Comp Pathol 2000;122:201-7.
45. Selvey L, Taylor R, Arklay A, Gerrard J. Screening of bat carers for antibodies to equine morbillivirus. Commun Dis Intell 1996;20:477-8.
46. Westbury HA, Hooper PT, Brouwer SL, Selleck PW. Susceptibility of cats to equine morbillivirus. Aust Vet J 1996;74:132-4.
47. Hyatt AD, Selleck PW. Ultrastructure of equine morbillivirus. Virus Res 1996;43:1-15.

49. Chua KB, Bellini WJ, Rota PA, Harcourt BH, Tamin A, Lam SK, et al. Nipah virus: a recently emergent deadly paramyxovirus. Science 2000;288:1432-5.
50. Chua KB, Goh KJ, Wong KT, Kamarulzaman A, Tan PS, Ksiazek TG, et al. Fatal encephalitis due to Nipah virus among pig-farmers in Malaysia. Lancet 1999;354:1256-9.
51. Goh KJ, Tan TC, Chew NK, Tan PS, Kamarulzaman A, Sarji SA, et al. Clinical features of Nipah virus encephalitis among pig farmers in Malaysia. N Engl J Med 2000;342:1229-35.
52. Middleton D, Westbury H, Morrissy C, van der Heide B, Russell G, Braun M, et al. Experimental Nipah virus disease in pigs: clinical features, virus excretion and subclinical infection. In: Cargill C, McOrist S, editors. Proceedings of the 16th International Pig Veterinary Society Congress. Ocean Grove, CA: International Pig Veterinary Society; 2000. p. 552.
53. Paton NI, Leo YS, Zaki SR, Auchus AR, Lee KE, Ling AE, et al. Outbreak of Nipah virus infection among abattoir workers in Singapore. Lancet 1999;354:1253-56.
54. Harcourt BH, Tamin A, Ksiazek TG, Rollin PE, Anderson LJ, Bellini WJ, et al. Molecular characterization of Nipah virus, a newly emergent paramyxovirus. Virology 2000;271:334-49.
55. Field HE,Young PL, Yob JM, Mills J, Hall L, Mackenzie JS. The natural history of Hendra and Nipah viruses. Microbes Infect 2001;3:307-14.
56. Medway L. The wild mammals of Malaya (Peninsular Malaysia) and Singapore. Kuala Lumpur: Oxford University Press, 1978.
57. Philbey AW, Kirkland PD, Ross AD, Davies RJ, Gleeson AB, Love RJ, et al. An apparently new virus (family Paramyxoviridae) infectious for pigs, humans, and fruit bats. Emerg Infect Dis 1998;4:269-71.
58. Chant K, Chan R, Smith M, Dwyer DE, Kirkland P. Probable human infection with a newly described virus in the family Paramyxoviridae. Emerg Infect Dis 1998;4:273-5.
59. Fraser G, Hooper P, Lunt R, Gould AR, Gleeson LJ, Hyatt AD, et al. Encephalitis caused by a lyssavirus in fruit bats in Australia. Emerg Infect Dis 1996;2:327-31.
60. Gould AR, Hyatt AD, Lunt R, Kattenbelt JA, Hengstberger S, Blacksell SD. Characterization of a novel lyssavirus isolated from Pteropid bats in Australia. Virus Res 1998;54:165-87.
61. Hooper P, Lunt R, Gould A, Samaratunga H, Hyatt AD, Gleeson LF, et al. A new lyssavirus—the first endemic rabies-related virus recognized in Australia. Bull Inst Pasteur 1997;95:209-18.
62. Lyssavirus Expert Group. Prevention of human lyssavirus infection. Commun Dis Intell 1996;20:505-7.
63. Allworth A, Murray K, Morgan J. A case of encephalitis due to a lyssavirus recently identified in fruit bats. Commun Dis Intell 1996;20:504.
64. Hanna JN, Carney IK, Smith GA, Tannenberg AE, Deverill JE, Botha JA, et al. Australian bat lyssavirus infection: a second human case, with a long incubation period. Med J Aust 2000;172:597-9.
65. McCall BJ, Epstein JH, Neill AS, Heel K, Field H, Barrett J, et al. Potential exposure to Australian bat lyssavirus, Queensland, 1996-1999. Emerg Infect Dis 2000;6:259-64.
66. Field H, McCall B, Barrett J. Australian bat lyssavirus infection in a captive juvenile black flying fox. Emerg Infect Dis 1999;5:438-40.
67. Hooper PT, Fraser GC, Foster RA, Storie GJ. Histopathology and immunochemistry of bats infected by Australian bat lyssavirus. Aust Vet J 1999;77:595-9.
68. Hayward JC, Gillespie SM, Kaplan KM. Packer R, Pallansch M, Plotkin S, et al. Outbreak of poliomyelitis-like paralysis associated with enterovirus 71. Pediatr Infect Dis J 1989;8:611-6.
69. Alexander JP, Baden L, Pallansch MA, Anderson LJ. Enterovirus infections and neurologic disease—United States, 1977-91. J Infect Dis 1994;169:905-8.
70. Cardosa MJ, Krishnan S, Tio PH, Perera D, Wong SC. Isolation of subgenus B adenovirus during a fatal outbreak of enterovirus 71-associated hand, foot and mouth disease in Sibu, Sarawak. Lancet 1999;354:987-91.
71. Komatsu H, Shimizu Y, Takeuchi Y, Ishiko H, Takada H. Outbreak of severe neurological involvement associated with enterovirus 71 infection. Pediatr Neurol 1999;20:17-23.
72. Lum LC, Wong KT, Lam SK, Chua KB, Goh AY, Lim WL, et al. Fatal enterovirus 71 encephalomyelitis. J Pediatr 1998;133:795-8.
73. Chang L-Y, Huang Y-C, Lin T-Y. Fulminant neurogenic pulmonary oedema with hand, foot and mouth disease. Lancet 1998;352:367-8.
74. Lum LCS, Wong KT, Lam SK, Chua KB, Goh AYT. Neurogenic pulmonary oedema and enterovirus 71 encephalomyelitis. Lancet 1998;352:1391.
75. Ho M, Chen E-R, Hsu K-H, Twu S-J, Chen K-T, Tsai S-F, et al. An epidemic of enterovirus 71 infection in Taiwan. N Engl J Med 1999;341:929-35.
76. Liu C-C, Tseng H-W, Wang S-M, Wang J-R, Su I-J. An outbreak of enterovirus 71 infection in Taiwan, 1998: epidemiologic and clinical manifestations. J Clin Virol 2000;17:23-30.
77. McMinn PC, Stratov I, Nagarajan L, Davis S. Neurological manifestations of enterovirus 71 infection in children during a hand, foot and mouth disease outbreak in Western Australia. Clin Infect Dis 2001;32:236-42.
78. Landry ML, Fonseca SS, Cohen S, Bogue CW. Fatal enterovirus type 71 infection: rapid detection and diagnostic pitfalls. Pediatr Infect Dis J 1995;14:1095-100.
79. Wang S-M, Liu C-C, Tseng H-W, Wang J-R, Huang C-C, Chen Y-J, et al. Clinical spectrum of enterovirus 71 infection in children in Southern Taiwan, with an emphasis on neurological complications. Clin Infect Dis 1999;29:184-90.
80. Yan J-J, Wang J-R, Liu C-C, Yang H-B, Su I-J. An outbreak of enterovirus 71 infection in Taiwan, 1998: a comprehensive pathological, virological and molecular study on a case of fulminant encephalitis. J Clin Virol 2000;17:13-22.
81. Huang C-C, Liu C-C, Chang Y-C, Chen C-Y, Wang S-T, Yeh T-F. Neurologic complications in children with enterovirus 71 infection. N Engl J Med 1999;341:936-42.
82. AbuBakar S, Chee H-Y, Al-Kobaisi MF, Xiaoshan J, Chua KB, Lam SK. Identification of enterovirus 71 isolates from an outbreak of hand, foot and mouth disease with fatal cases of encephalomyelitis in Malaysia. Virus Res 1999;61:1-9.
83. Shimizu H, Utama A, Yoshii K, Yoshida H, Yoneyama T, Sinniah M, et al. Enterovirus 71 from fatal and non-fatal cases of hand, foot and mouth disease epidemics in Malaysia, Japan, and Taiwan in 1997-1998. Jpn J Infect Dis 1999;52:12-5.
84. Brown BA, Oberste MS, Alexander JP, Kennett ML, Pallansch MA. Molecular epidemiology and evolution of enterovirus 71 strains isolated from 1970 to 1998. J Virol 1999;73:9969-75.
85. Wang J-R, Tsai H-P, Chen P-F, Lai Y-J, Yan J-J, Kiang D, et al. An outbreak of enterovirus 71 infection in Taiwan, 1998. II. Laboratory diagnosis and genetic analysis. J Clin Virol 2000;17:91-9.
86. World Health Organization. Revised 1999 HIV/AIDS estimates for the Western Pacific Region. STD/HIV/AIDS Surveillance Report. World Health Organization Regional Office for the Western Pacific Region, Manila. Issue Number 15, August 2000.
